# The role of athletic mental energy in the occurrence of flow state in male football (soccer) players

**DOI:** 10.1186/s13102-025-01090-w

**Published:** 2025-03-18

**Authors:** Yunus Emre Yarayan, Alexios Batrakoulis, Nuri Berk Güngör, Serkan Kurtipek, Kadir Keskin, Okan Burçak Çelik, Doğukan Batur Alp Gülşen, Gerasimos V. Grivas, Sameer Badri Al-Mhanna, Nouf H. Alkhamees, Bodor Bin Sheeha, Abdullah F. Alghannam

**Affiliations:** 1 Department of Physical Education and Sport Science, Siirt University, Siirt, Turkey; 2 Department of Physical Education and Sport Science, Democritus University of Thrace, Komotini, Greece; 3 Department of Physical Education and Sport Science, University of Thessaly, Trikala, Greece; 4 Department of Physical Education and Sport Science, Balıkesir University, Balıkesir, Turkey; 5 Department of Physical Education and Sport Science, Gazi University, Ankara, Turkey; 6 Department of Physical Education and Sport Science, Aydın Adnan Menderes University, Aydın, Turkey; 7 Physical Education and Sports, Division of Humanities and Political Sciences, Hellenic Naval Academy, Piraeus, Greece; 8 Department of Physiology, School of Medical Sciences, Universiti Sains Malaysia, Kubang Kerian, Kelantan, Malaysia; 9 Department of Rehabilitation Sciences, College of Health and Rehabilitation Sciences, Princess Nourah bint Abdulrahman University, P.O. Box 84428, Riyadh, 11671 Saudi Arabia; 10 Lifestyle & Health Research Center, Princess Nourah bint Abdulrahman University, Riyadh, Saudi Arabia

**Keywords:** Phychological performance, Mental resilience, Athletric focus, Sport cognition

## Abstract

**Background:**

This study examines the determinant role of athletic mental energy on the flow state in professional football (soccer) players. This research fills an important gap in the sports psychology literature by focusing on the interaction between mental energy and flow, a critical component of optimal performance.

**Methods:**

Accordingly, 254 male athletes (Mean age = 23.1 ± 5.4 years) voluntarily participated in the study. This study was designed with the relational survey model, one of the quantitative research models. Athletic Mental Energy Scale and Dispositional Flow State Scale-2 were used as data collection tools. After confirming the validity and reliability of the scales, the data were analyzed. In this study, blank data were first evaluated to check the suitability of the analysis and assumptions. After the structure of the scales was verified, firstly, when the skewness kurtosis values for the normality test were examined, it was deter-mined that the data were suitable for normal distribution as the values were between − 1.5 and + 1.5 and the Q-Q graph did not show deviations from the distribution. In this context, Pearson Correlation Analysis was used to determine the relationship between athletic mental energy and flow state, and structural equation modeling (SEM) analysis was used to determine the determinant role of mental energy. Mental energy significantly predicted the flow state (β = 0.81, *p* < 0.05), and the SEM results indicated a good model fit (χ²/df = 2.96, RMSEA = 0.08, GFI = 0.89, CFI = 0.90).

**Results:**

When the findings obtained from the research were analyzed, it was determined that athletic mental energy was a determinant of flow and contributed to 66% of the variance.

**Conclusion:**

As a result, it supports that athletes’ mental energy levels play an important role in their performance and contribute to the increase of optimal performance mood. These findings suggest that coaches and sports psychologists should focus on strategies to enhance athletes’ mental energy levels, such as incorporating mental skills training into their routines. The study emphasizes the importance of athletes’ ability to manage their mental energy levels and the development of strategies to increase optimal performance mood. By providing evidence for the link between mental energy and flow, this study contributes to advancing the understanding of performance optimization in professional sports.

## Introduction

In recent sports psychology studies, some psychological skills characterized by mental energy and flow are important for athletes to achieve the optimal performance range [1]. Indeed, researchers posit that an athlete’s performance in competitive sports is influenced not only by physical factors such as fitness, technique, and tactics, but also by mental and emotional characteristics [2]. Therefore, many studies have focused on psychological factors that positively or negatively affect athletic performance [3–8]. Based on these points, directing athletes’ athletic mental energy correctly and revealing their flow throughout the process can be characterized as the basic components of optimal performance [9, 10]. Despite this, limited empirical evidence exists on how mental energy directly influences the flow, particularly in professional football players, making this research essential for addressing this gap [11, 12].

The most important aspect of athletes who achieve success, especially in the sports environment is to focus on performance without losing focus of attention. Athletic mental energy, which is considered an exogenous variable within the scope of the research, enables athletes to maintain their physical and mental state continuously in training and competitions [13]. Athletic mental energy is a determinant for sport performance [13, 14]. However, the specific mechanisms through which mental energy interacts with flow remain underexplored, highlighting the need for further investigation into this relationship. In this context, although it appears as a concept that should be given importance in the sport psychology literature [15–19], researchers state that there is a need to investigate the factors that may affect the concept of mental energy [13, 20].

In the related literature, Lykken [21] states that it is important to have high mental energy levels for sports activities that require increased focus. Suinn [22] said that with this skill, athletes have self-confidence, they can focus and accordingly, they exhibit strong performances. Murphy [14] proposes that athletes’ performances are based on an energy pyramid structure, states that physical energy is at the bottom of this pyramid and emotional, mental and spiritual energy levels rise above the physical energy layer. This hierarchical structure emphasizes that mental energy, when effectively regulated, can amplify overall athletic performance by bridging physical and psychological resources. In the literature, mental energy has been conceptualized by researchers as an athlete’s perception of the current energy state [23] and the current energy state of athletes characterized by motivation, confidence and concentration intensity [13]. However, Nideffer [24], who gave a different explanation to the concept of mental energy, described this energy as a psychological skill that affects self-confidence, energy gathering and focus.

Mental energy [13], which is affected by internal and external sources, is associated with abstract thinking, creativity, self-regulation and self-awareness, which are described as high-level functions [13]. Mental energy, flow, and mindfulness [6], which are the focus of attention of researchers from different fields in the field of sport psychology, courage and psychological skills [25], eating behaviors [26], reaction time [27], attention ability [27, 28, 29, 30] and emotional experiences [31, 32]. However, studies integrating mental energy and flow within the dynamic and competitive context of team sports, particularly football, are scarce. This study aims to contribute to this gap by providing empirical evidence specific to this population.

The concept of flow, which is addressed within the scope of the research, is accepted as a functional state underlying optimal performance in sports, as it is a mental state that involves focusing away from distracting or negative thoughts in order to maintain performance in training or competition [33]. In the related literature, it is stated that flow is a source of mental energy as it is related to psychological factors such as performance and positive subjective experience [34, 35], self-concept [36] and attentional focus [37]. However, in the flow, which is called the optimal experience, the individual’s attention is completely focused on the current activity and the influence of external factors is minimized. It is stated that it is important for the individual to have a high level of mental energy to reach the flow [37]. As a matter of fact, it has been determined that individuals experiencing a state of flow have high mental energy and have more motivation, concentration and creativity. This shows that the flow can increase the level of mental energy of the individual and thus improve overall mental performance [38].

When factors such as stimuli, intense pressure, level of competition, team relationships, athlete injuries, coach and athlete relationships that affect performance in the sports environment are evaluated, our attention and balance need to be structured by external demands. Indeed, Nicholls et al. [39], state that in order to achieve the optimal performance range, the smallest developments can have significant effects on the outcome. In this context, these effects point to the continuity of mental energy, which has many features of the flow characteristic. Given the growing complexity of competitive sports, understanding how mental energy can be sustained and utilized effectively within the framework of flow theory becomes increasingly critical for both researchers and practitioners. In this context, it is important to make mental energy sustainable by coaches and athletes due to its connections with psychological concepts. In light of the information given above, it is seen that there is limited literature on mental energy and flow experience [6], which has a theoretical background in the literature.

In football, mental energy is a key factor that influences players’ ability to remain focused, make quick decisions, and react effectively under demanding conditions. Research suggests that mental fatigue can impair decision-making, reaction time, and technical execution in soccer players [40]. For instance, midfielders constantly assess their surroundings, anticipate passing lanes, and adapt to shifting game dynamics. Strong mental energy enables them to process information rapidly and execute strategic plays with precision, a skill essential for maintaining high performance levels [41]. Similarly, defenders must stay mentally engaged to time their tackles correctly and respond to sudden offensive moves, as cognitive fatigue has been shown to reduce reaction speed in defensive scenarios [42]. Even goalkeepers, who face high-pressure moments, rely on mental energy to recover quickly from mistakes and sustain their confidence throughout a match [43]. These examples demonstrate how mental energy contributes to different aspects of football performance, reinforcing the need for further research on its role in achieving an optimal flow state in competitive environments.

The aim of the present study was to determine the role of athletic mental energy in achieving the flow during sports performance in football players. Unlike previous research, this study utilizes structural equation modeling (SEM) to examine the interaction between latent variables such as mental energy and flow, offering a comprehensive analysis of their dynamic relationship in competitive football contexts. A novel aspect of this research lies in its emphasis on specific environmental and psychological factors within football that shape the relationship between mental energy and flow. This nuanced approach provides a deeper understanding of how these variables interact in a high-pressure competitive setting. Ultimately, this study seeks to bridge theoretical understanding and practical application, offering actionable strategies for enhancing mental energy and achieving optimal performance in sports contexts.

## Materials & methods

### Study design

A relational survey model was utilized in this study to examine the relationship between athletic mental energy and flow state. This model is designed to reveal the relationships between two or more variables based on a theoretical framework established through a detailed literature review. Although self-reported measures were used to assess these variables, the relational survey model, combined with SEM, enabled us to statistically test the hypothesized relationships between latent variables. This method provided a comprehensive understanding of the theoretical constructs and their interconnections, aligning the methodology with the study’s objectives [44]. The theoretical model constructed as a result of the literature review was tested with the structural equation model. Byrne [45] defines the structural equation model as “a combination of regression and factor analysis and a theoretical construct represented by latent and observed variables”. The relationship between athletic mental energy and flow state was analyzed using SEM. Unlike traditional regression analysis, SEM enables the modeling of relationships between latent variables—constructs that are not directly observed but inferred from multiple observed indicators. SEM offers several advantages, including the ability to account for measurement error and test the overall fit of the theoretical model. This approach allows for a more comprehensive understanding of the underlying relationships compared to linear regression, which analyzes only directly observed variables and does not provide model fit indices.” In addition, the main purpose of structural equation models is to statistically test a theoretical model with the data obtained and to determine how well the theory and research findings match [46].

### Participants

Within the scope of the research, G*Power multiple regression analysis was used to determine the strength of the sample size. As a result of the analysis, it was determined that 215 participants would be needed to obtain an effect size of 1-β = 95%, Alpha = 0.05, f2 = 0.10. In line with Suresh and Chandrashekara’s [47] suggestion to increase the sample size by approximately 10% due to the possibility of data withdrawal or missing data, the research group was composed of 254 athletes. The study consisted of 254 male participants who continued their sportive careers as licensed in the football branch with a professional sportsmanship level in 2022. While the average age of the participants in the current study was 23.1 ± 5.4 years, the average duration of their license was 6.6 ± 4.4 years.

### Data collection

First of all, in order to evaluate the ethical suitability of the research, Siirt University Ethics Committee was applied, and Ethics Committee Approval was obtained (30.06.2022-2957). The data collection process continued for 9 weeks. In this process, 25 coaches were contacted, and positive feedback was received from 22 of them. Due to the difficulty in contacting the coaches, snowball sampling, one of the purposive sampling methods, was used [48]. In this context, information about the purpose of the study was presented by the responsible researcher on the appointment dates. In the next stage, the personal information form was first applied to 195 athletes who could be reached face-to-face. Then, the mental energy and optimal performance mood scale were applied. Within the scope of the research, 59 participants were reached online via Google Forms.

### Personal information form

It consists of questions including demographic information about the gender, age, years of sport and competition category experience of the athletes included in the current study.

## Instruments

### Athletic mental energy scale

The scale developed by Lu et al. [13] to determine athletes’ perceptions of their athletic mental energy was adapted to Turkish by Yıldız et al. [19]. The scale consists of 6 sub-dimensions and 18 items on a 6-point Likert scale. In the adaptation of the scale, it was found that the 18-item fit index values were at an acceptable level and the factor structure was supported for the Turkish version. The highest total score (108) indicates that the individual has a high level of mental energy, while the lowest total score [18] indicates that the individual has a low level of mental energy [19].

### Dispositional flow Scale-2

The scale developed by Jackson and Eklund [49] was introduced into Turkish literature by Aşçı et al. [50] Internal consistency coefficients of DFS-2 were found ranging from 0.55 to 0.87. The scale consists of 9 sub-dimensions and 36 items and has a 5-point Likert scale. In the adaptation of the scale, it was seen that the 34-item fit index values were at an acceptable level and the factor structure was supported for the Turkish version. The highest total score (170) indicates that the individual experiences the optimal performance emotional state, while the lowest total score [34] indicates that the individual cannot experience the optimal performance emotional state [50].

### Confirmatory factor analysis results of the measurement tools used in the study

Confirmatory factor analysis was applied to test the construct validity of the scales used in this study. The goodness of fit values resulting from the measurement model are given in Table 1. The final fit index values obtained show that the structure of the scales used is confirmed [51].

**Table 1:** Confirmatory factor analysis results of the measurement tools used in the study

Model Fit Index	Perfect Range	Acceptable Range	Mental Energy	Flow
X^2^/sd	0 < X^2^/sd < 2	2 < X^2^/sd < 5	3.88	3.35
RMSEA	0.00 < RMSEA < 0.05	0.05 < RMSEA < 0.10	0.08	0.08
GFI	0.90 < GFI < 1.00	0.85 < GFI < 0.90	0.90	0.86
CFI	0.95 < CFI < 1.00	0.90 < CFI < 0.95	0.91	0.90

Χ^2^/sd: Chi-square to degrees of freedom; RMSEA: Root Mean Square Error of Approximation; GFI: Goodness of Fit Index; CFI: Comparative Fit Index.

### Statistical analysis

The analyses within the scope of the research were carried out through AMOS 22.0 and SPSS 22.0 programs. In the data analysis process, it was tested whether the data set met the normal distribution conditions. Skewness and kurtosis values were analyzed for normality assumptions. It was determined that the values of skewness (Mental Energy: -0.43; Flow: -0.10) and kurtosis (Mental Energy: -0.05; Flow: 0.15) ranged between − 1.5 and + 1.5 [52]. Then, firstly, Person-Correlation analysis was used to determine the relationships between the variables and the theoretical model was tested with SEM analysis. Correlation analysis was used. The coefficients obtained as a result of this analysis were evaluated according to Schober et al. [53] (0.00-0.10: insignificant, 0.10–0.39: weak, 0.40–0.69: moderate, 0.70–0.89: strong, 0.90-1: very strong). Robust Maximum Likelihood (MLR) method was used to calculate the measurement and SEM analysis. In this study, x^2^/df, Comparative Fit Index (CFI), Goodness of Fit Index (GFI), Root Mean Square Error Approximation (RMSEA) values recommended by Kline [51] for SEM studies were reported.

## Results

In Table 2, the mean scores of the participants mean scores for the vigor sub-dimension (x̄ = 5.01), confidence sub-dimension (x̄ = 4.57), motivation sub-dimension (x̄ = 5.28), fatigue sub-dimension (x̄ = 4.15), concentration sub-dimension (x̄ = 3.93), calm sub-dimension (x̄ = 4.20) and the whole scale (x̄ = 4.55) were determined.

**Table 2:** Participants’ mean scores from the athletic mental energy scale

Mental Energy	*N*	Min	Max	$\bar x$	S
Vigor	254	1.33	6.00	5.01	0.78
Confidence Motivation Tireless Concentration Calm	254 254 254 254 254	2.00 2.33 1.00 1.00 1.00	6.00 6.00 6.00 6.00 6.00	4.57 5.28 4.15 3.93 4.20	0.81 0.72 1.13 0.92 1.05
Total	254	2.61	6.00	4.55	0.64

N: number of participants; Min: minimum; Max: maximum; $\bar x$: mean; S: standard deviation

The examination of the relationship between variables with Pearson product-moment correlation showed that there is a moderate and positive relationship between athletic mental energy and trait dispositional flow (*r* = 0.633, *p* < 0.05). After revealing the relationships between the variables of the study, the predictive effect of athletic mental energy on trait dispositional flow was tested with SEM analysis and presented in the following section.

In Table 3, it can be said that the model tested according to Kline [51] meets the required goodness of fit criteria (x^2^/sd = 2.96, RMSEA = 0.08, GFI = 0.89, CFI = 0.90). While the overall fit indices indicate an acceptable fit, some metrics, such as GFI and RMSEA, are at the lower bounds of the acceptable range. This suggests that while the model adequately represents the data, certain refinements could improve its fit further. For instance, incorporating additional observed variables or reevaluating latent variable relationships might enhance the model’s overall fit and predictive power. Although the scale used in this study consists of multiple sub-dimensions, a second-order confirmatory factor analysis (DFA) was conducted to evaluate these sub-dimensions under a single higher-order construct. This approach aligns with the theoretical framework of the study, which posits that the sub-dimensions collectively represent an overarching latent variable (e.g., athletic mental energy or flow). The decision to use a second-order model was based on the strong theoretical and empirical evidence supporting the conceptual coherence of the sub-dimensions as facets of a unified construct. Additionally, this modeling approach simplifies the interpretation of results while maintaining the integrity of the sub-dimensions within the broader latent variable. After examining the goodness-of-fit index values, the paths in the model and the parameter estimates were analyzed in detail. The slight deviation in GFI (0.89) from the ideal range (≥ 0.90) and the RMSEA value (0.08), which lies at the threshold of acceptability, highlight potential areas for optimization. These include refining the measurement scales or increasing the sample size to improve variability and robustness. While integrating additional observed variables might enhance model fit, the second-order model effectively captures the relationships between the constructs of interest, as reflected in the acceptable fit indices. Despite these considerations, the current indices provide substantial support for the proposed theoretical framework, validating the hypothesized relationships between athletic mental energy and flow. The findings confirm the structural model’s adequacy in explaining these relationships, offering a valuable contribution to the field of sports psychology.Future studies could adopt alternative modeling approaches, such as multigroup analysis or longitudinal designs, to further validate these findings across diverse populations and competitive settings. Such methods could also overcome limitations associated with cross-sectional data, enabling a deeper exploration of the dynamic and reciprocal relationship between mental energy and flow. According to the model, the standardized β coefficients, standard error, critical ratio, p, and R² values between the variables are shown in Table 4. These values offer further insights into the strength and significance of the relationships identified in this study.

**Table 3:** Structural equation model fit index values

Model Fit Index	Perfect Range	Acceptable Range	SEM
X^2^/sd	0 < X^2^/sd < 2	2 < X^2^/sd < 5	2.96
RMSEA	0.00 < RMSEA < 0.05	0.05 < RMSEA < 0.10	0.08
GFI	0.90 < GFI < 1.00	0.85 < GFI < 0.90	0.89
CFI	0.95 < CFI < 1.00	0.90 < CFI < 0.95	0.90

Χ^2^/sd: Chi-square to degrees of freedom; RMSEA: Root Mean Square Error of Approximation; GFI: Goodness of Fit Index; CFI: Comparative Fit Index; SEM: Structural Equation Modeling

**Fig. 1:**
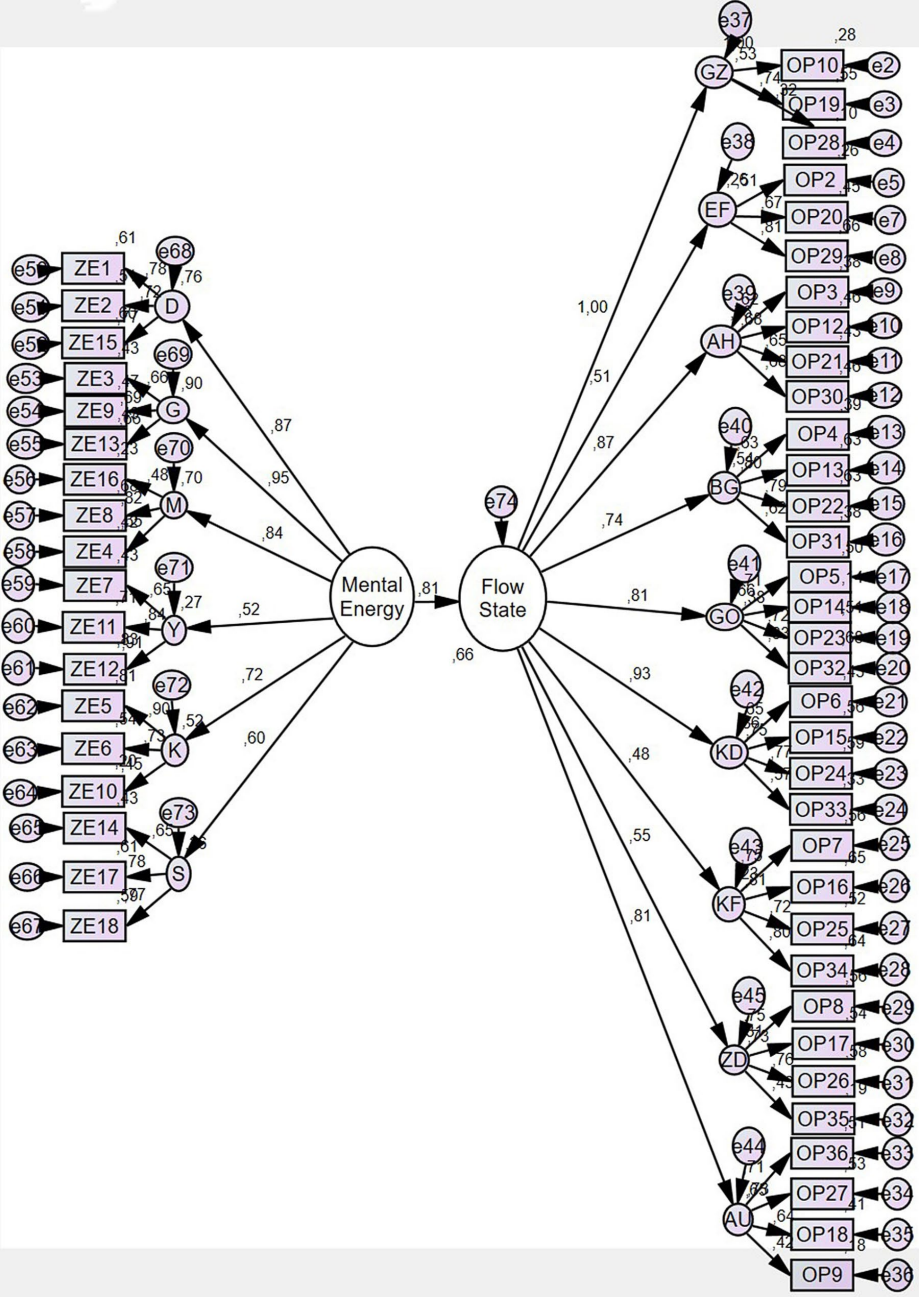
Structural equation model

In Table 4, the results of the analysis of the theoretical model tested indicate that there is a statistically significant effect on the relationship between athletic mental energy and dispositional flow, (β1 = 0.81; *p* < 0.05). From this point of view, the hypothesis of the study is accepted. When the Squared Multiple Correlations (R^2^) value of the model was examined, it was determined that athletic mental energy explained 66% of the dispositional flow. In addition, when the beta coefficients were examined as a result of the analysis, the standardized regression coefficient (β) of athletic mental energy was found to be 0.81. This result can be interpreted as the increase in the level of athletic mental energy will increase the dispositional flow. The path diagram of the model is presented in Fig. 1.

**Table 4:** Structural equation model results

Variables		β	Standard Err	Critical Ratio	*p*	*R* ^2^
Mental Energy	→Flow	0.81	0.046	12.5	<0.05	0.66

β: Standardized beta coefficient; Std. Err: Standard Error; Critical Ratio (CR): z value, indicating the ratio of the estimate to its standard error; R²: Explained variance are reported

## Discussion

This study focuses on psychological characteristics that play an important role in athletic performance. For athletes to be successful, they need to be both physically and mentally ready. This research reveals that athletes’ mental energy levels affect their dispositional flow. The results show that athletes with higher mental energy levels have a higher optimal performance mood. These findings support that mental energy is an important factor in sport performance.

The research conducted by Öner [6], which is the only study in the relevant literature, in which the effect of athletic mental energy on the flow is revealed, supports our findings. In a study conducted by Johnson et al. [54], it was found that mental energy can affect cognitive performance and that cognitive functions improve with increasing energy levels. Similarly, another study by Brown and Bray [55] found that mental energy affects sports performance and that athletes with high energy levels perform more effectively. Likewise, Yarayan et al. [56] found that imagery and emotional intelligence are important factors for sports performance in elite middle-and long-distance runners. Lane et al. [57] examined the ability of athletes to manage mental energy and found that they were able to increase their mental energy by using strategies to manage their pre-performance emotional states and this had positive effects on performance. These studies highlight that specific sub-dimensions of mental energy, such as concentration and confidence, play critical roles in achieving the flow. For example, concentration helps athletes maintain focus on the task at hand while minimizing distractions, whereas confidence boosts their belief in their ability to succeed, reinforcing their persistence in challenging situations. The findings of the current study align with these observations, emphasizing the multidimensional nature of mental energy and its unique contributions to flow. Recent research has further extended the understanding of athletic mental energy, demonstrating that it not only affects performance but also contributes to psychological well-being. A study exploring athletic mental health’s mediating role between performance and well-being found that both the affective and cognitive components of athletic mental health influence this relationship. The study, conducted, across various sports disciplines and levels of competition, also indicated the athletic mental energy plays a key role in preserving athletes’ psychological well-being post-competition, offering valuable insights into how mental energy can support long-term athlete health and performance [12]. Together with these studies, it is seen that studies are focusing on the variables of mental energy on health [58], motivation [59], emotional state [60], life stress and burnout [17], quality of life [61]. These findings suggest that mental energy plays a critical role in sustaining athletes’ well-being, especially post-competition, and offers long-term benefits for maintaining optimal performance.

Considering the characteristics that have been examined in relation to optimal performance mood; emotional intelligence [62], goal orientation and thrill seeking [63], personality traits and perfectionism [64]. Considering the research findings, another variable that helps to explain the optimal performance mood, which is handled with many variables, is athletic mental energy. Athletic mental energy is a feature that helps athletes to perform positively [22]. In this context, it is expected to be related to the affective goal orientation that leads the athlete to success, and it is supported by the research findings. Practical strategies for enhancing mental energy could include mental skills training programs focusing on concentration improvement, relaxation techniques, and self-confidence building exercises. For example, visualization techniques and mindfulness practices could help athletes maintain high energy levels during competition or recover more effectively post-performance. Coaches and sports psychologists could integrate these methods into regular training routines to optimize both individual and team performance. From this point of view, in order to increase both individual and team performance of athletes, it is important to include athletic mental energy-oriented studies in training in order to protect their optimal performance emotional states from negative factors. In addition, the development of athletic mental energy skills will positively affect the optimal performance emotional state. When the results of the model created for the purpose of the study are examined, it is seen that athletic mental energy explains 66% of the continuous optimal performance mood. Therefore, it can be stated that mental energy has a significant effect on the orientation of all senses and emotions toward the same goal. With this research, this deficiency in the literature has been eliminated.

An important point to keep in mind is that the effect of athletic mental energy on flow can vary from person to person and situation to situation. Each athlete’s level of athletic mental energy may differ. In this context, athletes may need different mental skills for optimal performance. Therefore, athletes need to recognize, manage and develop their mental energy individually. Tailored interventions based on an athlete’s specific mental energy profile could be a valuable tool in optimizing performance. For example, an athlete with strong concentration but lower confidence might benefit more from confidence-building exercises, while another with high energy levels might focus on sustaining these levels through recovery strategies. In conclusion, athletic mental energy is an important factor for achieving flow and improving performance. For athletes, managing their mental energy and supporting the flow is a critical step on the path to success. This study shows that mental energy influences optimal performance mood based on research results on athletes. The findings that mental energy plays an important role in sports performance emphasize that it should be taken into account in the mental and physical preparation processes of athletes.

The current study has a few limitations. Firstly, the findings are limited to team athletes and male participants. Therefore, it is important to include female athletes in future research. Secondly, the research sample consists of team athletes. This approach has the limitation of not providing an in-depth view of a specific branch. In future studies, it is recommended to focus on branch groups separately in order to reveal subjective results when the relevant characteristics are considered.

In the present study, instantaneous or one-time measurements were made to evaluate the relationship between mental energy and optimal performance mood. Since monitoring changes over time will provide a better understanding of the relationship, it is recommended that future research should be planned longitudinally. In addition, by creating experimental groups on athletes with different mental energy levels, how specific mental energy levels affect performance and flow can be evaluated more precisely in future research.

### Strengths, limitations, and future directions

#### Strengths

This study makes significant contributions to the understanding of the relationship between mental energy and flow in the field of sports psychology. By focusing on the sub-dimensions of mental energy—such as concentration, confidence, and motivation the study provides a nuanced perspective on how these components influence optimal performance mood. The model explains 66% of the variance in continuous optimal performance mood, demonstrating the strong explanatory power of athletic mental energy. Additionally, the study bridges the gap between theory and practice by offering actionable insights for coaches, sports psychologists, and athletes to enhance individual and team performance.

#### Limitations

Despite its contributions, the study has certain limitations:

1. Participant Diversity: The sample includes only male team athletes, which limits the generalizability of the findings to female athletes, amateur players, or individual sports.
2. Cross-Sectional Design: The cross-sectional nature of the study restricts the ability to understand how the relationship between mental energy and flow evolves over time. This design also limits the establishment of causal relationships between variables.
3. Focus on Team Sports: The exclusive focus on team sports does not provide an in-depth view of how mental energy impacts performance in specific individual sports or different competitive contexts.

#### Future directions

To address these limitations and build on the findings of this study, future research could consider the following:

1. Inclusion of Diverse Populations: Future studies should explore the relationship between mental energy and flow in female athletes, amateur players, and athletes from individual sports to enhance the generalizability of the findings.
2. Longitudinal Studies: Research designs that monitor changes in mental energy and flow over time could provide deeper insights into the dynamics of these variables and establish causal relationships.
3. Experimental Interventions: Studies focusing on interventions designed to enhance mental energy such as mindfulness training, visualization techniques, or relaxation exercisescould provide evidence-based strategies for improving performance and flow.
4. Comparative Analyses Across Sports: Investigating differences between team and individual sports could uncover sport-specific mechanisms that influence mental energy and flow, contributing to tailored strategies for athletes in diverse disciplines.

## Conclusion

The findings of this study underscore the pivotal role of mental energy in achieving flow and optimal performance. By highlighting the multidimensional nature of mental energy and its influence on flow, this research offers a foundation for future studies to explore these constructs further. Practically, the study provides valuable guidance for coaches, sports psychologists, and athletes in developing strategies to enhance mental energy levels and sustain optimal performance. Ultimately, this research reinforces the importance of integrating mental energy management into both training and competition preparation, contributing to long-term success in sports psychology and performance enhancement.

## Data Availability

The datasets generated and/or analysed during the current study are not publicly available but are available from the corresponding author on reasonable request.
